# Cardiovascular Toxicities of Breast Cancer Treatment: Emerging Issues in Cardio-Oncology

**DOI:** 10.3389/fonc.2015.00066

**Published:** 2015-03-18

**Authors:** Sharad Goyal, Bruce George Haffty

**Affiliations:** ^1^Department of Radiation Oncology, Rutgers Cancer Institute of New Jersey, New Brunswick, NJ, USA; ^2^Rutgers Robert Wood Johnson Medical School, New Brunswick, NJ, USA

**Keywords:** cardiovascular disease, radiation therapy, chemotherapy, breast cancer, toxicities

In 2015, the American Cancer Society estimates that 234,000 new cases of breast cancer will be diagnosed along with an additional 60,000 cases of carcinoma *in situ*. Nearly 40,000 women will die due to breast cancer annually (1). Current management options for Stage 0, I, & II breast cancer include mastectomy, breast conserving surgery (BCS), or breast conserving surgery followed by whole breast radiation therapy (BCS + RT); the use of chemotherapy is independent of local therapy. Coronary artery disease (CAD) and cerebrovascular disease (CVD) are the first and third leading causes of death, respectively, among men and women in the United States (2). Patients with CVD share multiple common risk factors and lifestyle behaviors in addition to frequently suffering from multiple comorbid conditions. Tobacco use, hypertension, high cholesterol, diabetes, physical inactivity, and poor nutrition are all established risk factors of heart disease. The carcinogenic potential of antihypertensive medications has been widely debated and while a meta-analysis found no increase in cancer incidence or mortality, they could not rule this out with certain combinations of drugs (3). In addition, as antihypertensive medications for cardiovascular disease improve, patients are living longer and are at greater risk for developing cancer. Moreover, patients with diseases such as breast cancer may develop CVD from treatment, such as use of chemotherapy and radiation therapy (RT). Inherently within these competing risks of morbidity and mortality lays the intersection of the disciplines of cardiology and oncology.

Cardio-oncology is a relatively new field which offers an interdisciplinary and integrative management approach to cancer patients with cardiovascular risks specifically designed to mitigate risks of oncologic therapies and provide early detection and treatment of those at greatest risk of cardio-toxicity. Given the growing clinical relevance of cardio-oncology, this *Frontiers in Oncology Research Topic* is focused on providing information to practitioners in this quickly growing discipline. In this research topic, Mookadam et al. eloquently frames the current practice in cardio-oncology and provides future strategy and direction for this novice field.

Effects on the heart are a potentially significant and serious clinical problem in the treatment of breast cancer with RT. Over the course of the past 50 years, there have been great advances in the delivery of RT due to the development of new techniques, beam energy, improvement in imaging modalities, and development of image registration strategies. In this research topic, Yue et al. and Chen et al. present novel techniques to track cardiac motion during radiation treatments using fluoroscopy and cardiac MRI, respectively (4, 5). In addition, Beck et al. reviews the various radiotherapy treatment techniques used in breast cancer patients to reduce cardiac dose (6). The study reported by Merino Lara et al. attempts to translate the mean cardiac dose for various breast radiotherapy techniques to the risk of a major cardiovascular event (7). Finally, an editorial by Khan and colleagues expressively frames the debate of cardiac irradiation into perspective (8).

It is hypothesized that cardiac damage from RT is correlated to the dose absorbed by the heart and differs between left- and right-breast radiotherapy. The damage to cardiac micro- and macro-vasculature is the pathophysiological cause of RT-related heart disease. Taunk and colleagues summarize the literature regarding the underlying pathologic abnormalities and mechanisms of RT-related heart disease (9).

Other manuscripts in this issue reflect the eclectic nature of the field of cardio-oncology. Sharp and George review the potential benefit of stem cell therapy against cardio-toxicities from breast cancer treatments, while Tian et al. review the literature of serum biomarkers of cardiac toxicity after breast cancer treatments (10, 11). Finally, Guo and Wong discuss cardiovascular toxicities of chemotherapy and targeted therapies used in the systemic treatment of breast cancer (12).

It is our hope that through this research topic, we may continue the dialog of reducing collateral damage to the cardiovascular system by breast cancer therapies, both local and systemic. Guidelines regarding the prevention and treatment of cardiac toxicities are scant and collaborative efforts are needed to facilitate their development.

## Conflict of Interest Statement

The authors declare that the research was conducted in the absence of any commercial or financial relationships that could be construed as a potential conflict of interest.
